# A Single Pair of Neurons Modulates Egg-Laying Decisions in *Drosophila*

**DOI:** 10.1371/journal.pone.0121335

**Published:** 2015-03-17

**Authors:** Chia-Lin Wu, Tsai-Feng Fu, Yen-Yun Chou, Sheng-Rong Yeh

**Affiliations:** 1 Department of Biochemistry, College of Medicine, Chang Gung University, Taoyuan, Taiwan; 2 Graduate Institute of Biomedical Sciences, College of Medicine, Chang Gung University, Taoyuan, Taiwan; 3 Department of Medical Research, Chang Gung Memorial Hospital, Taoyuan, Taiwan; 4 Department of Applied Chemistry, National Chi-Nan University, Nantou, Taiwan; 5 Department of Biomedical Sciences, College of Medicine, Chang Gung University, Taoyuan, Taiwan; Alexander Fleming Biomedical Sciences Research Center, GREECE

## Abstract

Animals have to judge environmental cues and choose the most suitable option for them from many different options. Female fruit flies selecting an optimum site to deposit their eggs is a biologically important reproductive behavior. When given the direct choice between ovipositing their eggs in a sucrose-containing medium or a caffeine-containing medium, female flies prefer the latter. However, the neural circuits and molecules that regulate this decision-making processes during egg-laying site selection remain poorly understood. In the present study, we found that amnesiac (*amn*) mutant flies show significant defects in egg-laying decisions, and such defects can be reversed by expressing the wild-type *amn* transgene in two dorsal paired medial (DPM) neurons in the brain. Silencing neuronal activity with an inward rectifier potassium channel (*Kir2*.*1*) in DPM neurons also impairs egg-laying decisions. Finally, the activity in mushroom body αβ neurons is required for the egg-laying behavior, suggesting a possible “DPM-αβ neurons” brain circuit modulating egg-laying decisions. Our results highlight the brain circuits and molecular mechanisms of egg-laying decisions in *Drosophila*.

## Introduction

*Drosophila* females selecting a suitable site to deposit their eggs is a biologically important behavior that allows the study of a simple decision-making process [1,2,3,4]. Female flies have to judge and select a proper site for laying eggs to ensure that the environment is optimal for survival of their offspring [1,2,4]. The specific molecular mechanisms and neural circuits that control egg-laying decisions in *Drosophila* are poorly understood

The *amnesiac (amn)* gene encodes a neuropeptide (AMN) whose function was first identified in the context of olfactory associative memory in *Drosophila* [5]. An *amn* mutant can associate specific odors with an electrical foot shock or a sugar reward, but will forget this information quickly, which suggests that *amn* is specifically involved in memory instead of initial learning [5,6,7]. A remarkable study demonstrates that the *amn* gene product is strongly expressed in two dorsal paired medial (DPM) neurons that innervate all lobes of the mushroom body [6]. Targeted expression of the *amn* gene in two DPM neurons rescues olfactory associative memory in an *amn* mutant background [6]. The *Drosophila* mushroom body is a paired neuropil structure crucial for olfactory associative memory and can be structurally divided into the αβ, α´β´, and γ neurons according to their axonal fiber distributions. The dendrites of the mushroom body form a calyx and project their axons anteriorly to form the peduncle and extend to the αβ, α´β´, and γ lobes in the middle brain [8]. Numerous studies on the behavior and brain-anatomy of fruit flies have identified that the mushroom body is crucial for olfactory associative memory [9,10,11,12,13,14], sleep [15,16], and temperature-preference behavior [17,18].

In this study, we first identified that the *amn* gene product is essential for normal egg-laying decisions by analyzing a collection of *amn* mutant flies. In addition, genetically silencing the neuronal activity in DPM neurons disrupts this behavior. Targeting the expression of the *amn* transgene in two DPM neurons can fully reverse this behavioral defect in *amn* mutant flies. Finally, we demonstrated that neuronal activity in the mushroom body αβ neurons is required for normal egg-laying decisions suggesting that the possible “DPM-αβ neuron” circuit controls this behavior via AMN neuropeptide release.

## Results

### Aged flies exhibit normal egg-laying decisions

**Fig 1:**
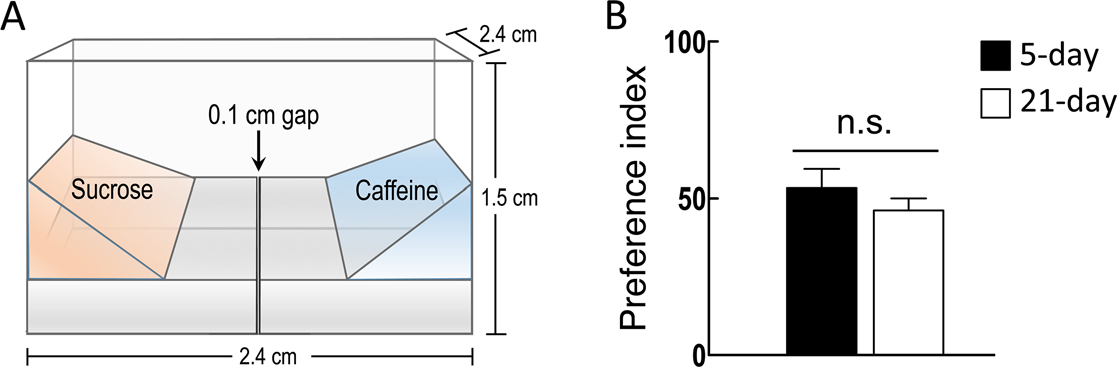
Aging does not alter *Drosophila* egg-laying decisions. (A) Schematic representation of the chamber for egg-laying decisions assays. Two 1% agarose media, containing either 100 mM sucrose (orange) or 100 mM caffeine (blue), were loaded above the 3% hard agarose medium (grey). A gap (0.1 cm) was made on the 3% agarose medium in the middle of the chamber. (B) Egg-laying decisions of wild-type flies at different ages (5-day and 21-day). Each value represents mean ± SEM (N = 36–42, n.s., not statistically significant).

It has been reported that female flies avoid laying eggs on a medium containing sucrose, and that this egg-laying site selection relies on a simple decision-based behavioral process [1]. We took advantage of this behavioral assay to explore the molecules and brain circuits underlying egg-laying decisions. A plastic egg-laying chamber was placed in a sweet (sucrose-containing) and a bitter (caffeine-containing) 1% soft agarose medium, and the two media were separated by a region of 3% hard agarose, which contained a small gap in the middle to prevent diffusion of sucrose or caffeine to the opposite site [2](Fig. 1A). Consistent with the previous findings, female flies prefer to deposit their eggs on a bitter substrate (Fig. 1B)[1]. Decision-making processes involve neuronal function in the brain, and aging significantly alters the functioning of the nervous system [19,20]. We first tested whether egg-laying decisions changed with aging. The 21-day old female flies were used for the behavioral assays [21], and we found that aged flies still executed normal egg-laying decisions compared to young flies (5-day old) (Fig. 1B).

### *amn* expression in two DPM neurons is essential for egg-laying decisions

**Fig 2:**
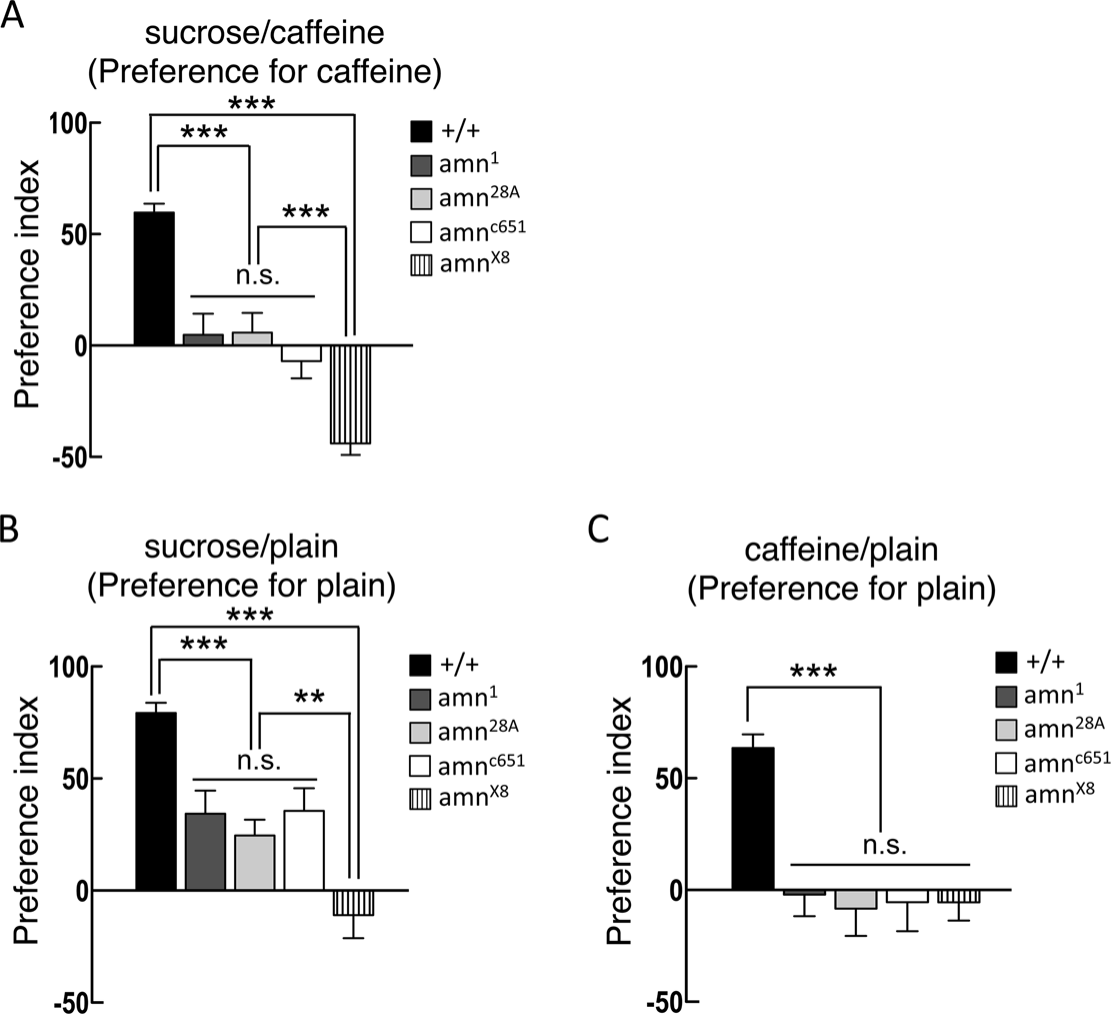
*amn* mutants show defects in egg-laying decisions. (A) In sucrose/caffeine chamber, *amn* ^*1*^, *amn* ^*28A*^, *amn* ^*c651*^, and *amn* ^*X8*^ flies showed significant difference in egg-laying decisions compared to wild-type flies. *amn* ^*X8*^ showed significant difference compared to the other *amn* mutants. Each value represents mean ± SEM (n = 34–35, ***P < 0.001). (B) In sucrose/plain chamber, *amn* ^*1*^, *amn* ^*28A*^, *amn* ^*c651*^, and *amn* ^*X8*^ flies showed significant difference in egg-laying decisions compared to wild-type flies. *amn* ^*X8*^ showed significant difference compared to the other *amn* mutants. Each value represents mean ± SEM (n = 20, ***P < 0.001, **P < 0.01). (C) In caffeine/plain chamber, *amn* ^*1*^, *amn* ^*28A*^, *amn* ^*c651*^, and *amn* ^*X8*^ flies showed significant difference in egg-laying decisions compared to wild-type flies. Each value represents mean ± SEM (n = 23–27, ***P < 0.001).

*amn* encodes a preproneuropeptide with limited similarity to pituitary-adenylyl-cyclase-activating peptide (PACAP) [22]. It has been reported that AMN plays a critical role in behaviors of *Drosophila* such as olfactory memory and sleep [5,7,23]. To examine the role of the *amn* gene in egg-laying decisions, a collection of *amn* mutants were analyzed for their egg-laying preference in the behavioral chambers. Interestingly, we found that *amn* ^*1*^, *amn* ^*28A*^, *amn* ^*c651*^, and *amn* ^*X8*^ mutants showed significant defects in egg-laying preference compared to wild-type flies (Fig. 2A). We further examined the egg-laying preference in the chamber containing sucrose or caffeine substrate in one side and a plain substrate in the opposite side. Consistent with the previous findings, wild-type female flies avoided laying eggs on sucrose (Fig. 2B) or caffeine (Fig. 2C) substrates when the other option was a plain substrate [1]. All the *amn* mutants show significant difference in egg-laying preference in sucrose/plain or caffeine/plain chambers compared to wild-type flies (Fig. 2B and 2C). These results indicate the *amn* gene is critical for egg-laying decisions in sucrose/caffeine, sucrose/plain, and caffeine/plain mediums.

**Fig 3:**
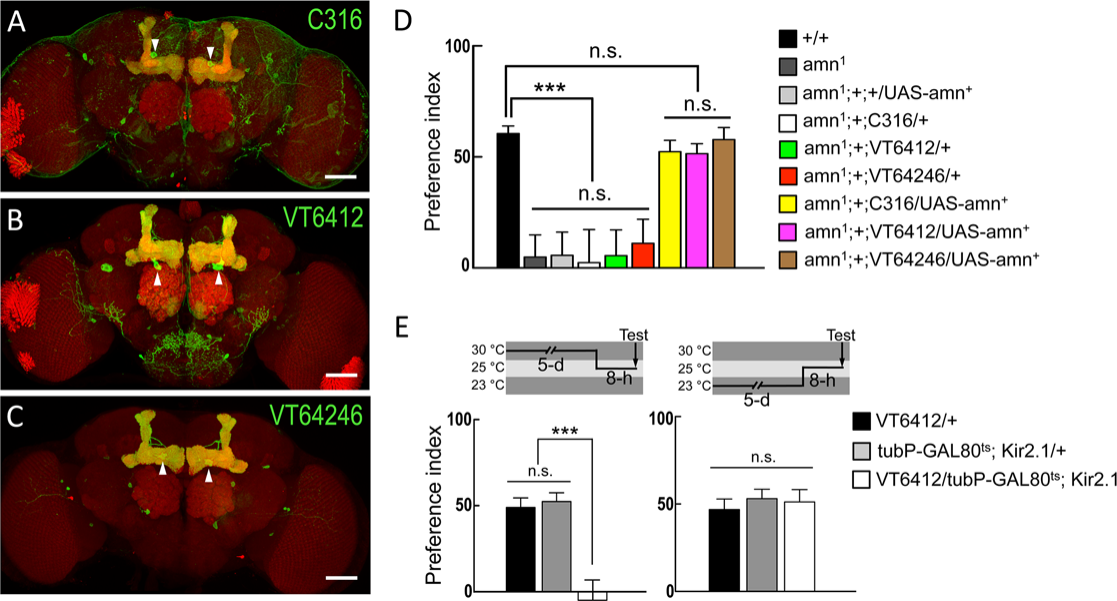
Expression of *amn* transgene in DPM neurons reverses the defects of egg-laying decisions in *amn* mutants. (A) The expression pattern of *C316-GAL4* (green). The brain was immunostained with DLG antibody (red). Arrowheads indicate the somata of DPM neurons. The scale bar represents 50 μm. Genotype was as follow: *UAS-mCD8*::*GFP/+; C316-GAL4/UAS-mCD8*::*GFP*. (B) The expression pattern of *VT6412-GAL4* (green). The brain was immunostained with DLG antibody (red). Arrowheads indicate the somata of DPM neurons. The scale bar represents 50 μm. Genotype was as follow: *UAS-mCD8*::*GFP/+; VT64246-GAL4/UAS-mCD8*::*GFP*. (C) The expression pattern of *VT64246-GAL4* (green). The brain was immunostained with DLG antibody (red). Arrowheads indicate the somata of DPM neurons. The scale bar represents 50 μm. Genotype was as follow: *UAS-mCD8*::*GFP/+; VT64246-GAL4/UAS-mCD8*::*GFP*. (D) Overexpression of the *amn* transgene (*amn* ^*+*^) in DPM neurons reversed the deficiency of egg-laying decisions in *amn* ^*1*^ background. Each value represents mean ± SEM (n = 22–24, ***P < 0.001, n.s., not statistically significant). Genotypes were as follows: (1) +/+, (2) *amn* ^*1*^ */amn* ^*1*^, (3) *amn* ^*1*^ */amn* ^*1*^ *; +/+; +/UAS-amn* ^*+*^, (4) *amn* ^*1*^ */amn* ^*1*^ *; +/+; C316-GAL4/+*, (5) *amn* ^*1*^ */amn* ^*1*^ *; +/+; VT6412-GAL4/+*, (6) *amn* ^*1*^ */amn* ^*1*^ *; +/+; VT64246-GAL4/+*, (7) *amn* ^*1*^ */amn* ^*1*^ *; +/+; C316-GAL4/UAS-amn* ^*+*^, (8) *amn* ^*1*^ */amn* ^*1*^ *; +/+; VT6412-GAL4/UAS-amn* ^*+*^, and (9) *amn* ^*1*^ */amn* ^*1*^ *; +/+; VT64246-GAL4/UAS-amn* ^*+*^. (E) Effects of acute silencing of neuronal activity in DPM neurons on egg-laying decisions. The schematics of the temperature shift protocols are shown above each graph. Each value represents mean ± SEM (n = 17–26, ***P < 0.001. n.s., not statistically significant). Genotypes were as follows: (1) *+/+; VT6412-GAL4/+*, (2) *tubP-GAL80* ^*ts*^ */+; UAS-Kir2*.*1/+*, and (3) *tubP-GAL80* ^*ts*^ */+; VT6412-GAL4/UAS-Kir2*.*1*.

Although the *amn* gene is expressed throughout the fly brain, targeting expression of the *amn* gene in two DPM neurons restores the olfactory memory in *amn* mutant flies [6]. We therefore tested whether the *amn* gene product in DPM neurons is involved in egg-laying decisions. We used a GAL4/UAS system to target expression of the wild-type *amn* transgene (*amn* ^*+*^) in DPM neuron by applying three independent DPM specific GAL4 drivers, the *C316-GAL4*, *VT6412-GAL4*, and *VT64246-GAL4* (Figs. 3A, 3B, and 3C). *amn* ^*1*^ is an EMS-induced mutation in the allele of the *amn* gene that causes a significant reduction in the *amn* transcript [24]. Therefore, we chose *amn* ^*1*^ to perform the following rescue experiment. Flies carrying the *amn* ^*1*^ */amn* ^*1*^ *; +/+; C316-GAL4/UAS-amn* ^*+*^, or *amn* ^*1*^ */amn* ^*1*^ *; +/+; VT6412-GAL4/UAS-amn* ^*+*^, or *amn* ^*1*^ */amn* ^*1*^ *; +/+; VT64246-GAL4/UAS-amn* ^*+*^ showed normal egg-laying preferences compared to wild-type flies, indicating that targeting expression of the *amn* transgene in DPM neurons restored typical egg-laying preference (Fig. 3D). In addition, acute silencing of the neuronal activity in DPM neurons by an inward rectifier potassium channel (*Kir2*.*1*) disrupts egg-laying preferences (Fig. 3E), suggesting a role of neurotransmission in DPM neurons for execution of normal egg-laying preference.

### Mushroom body αβ neurons are required for egg-laying decisions

**Fig 4:**
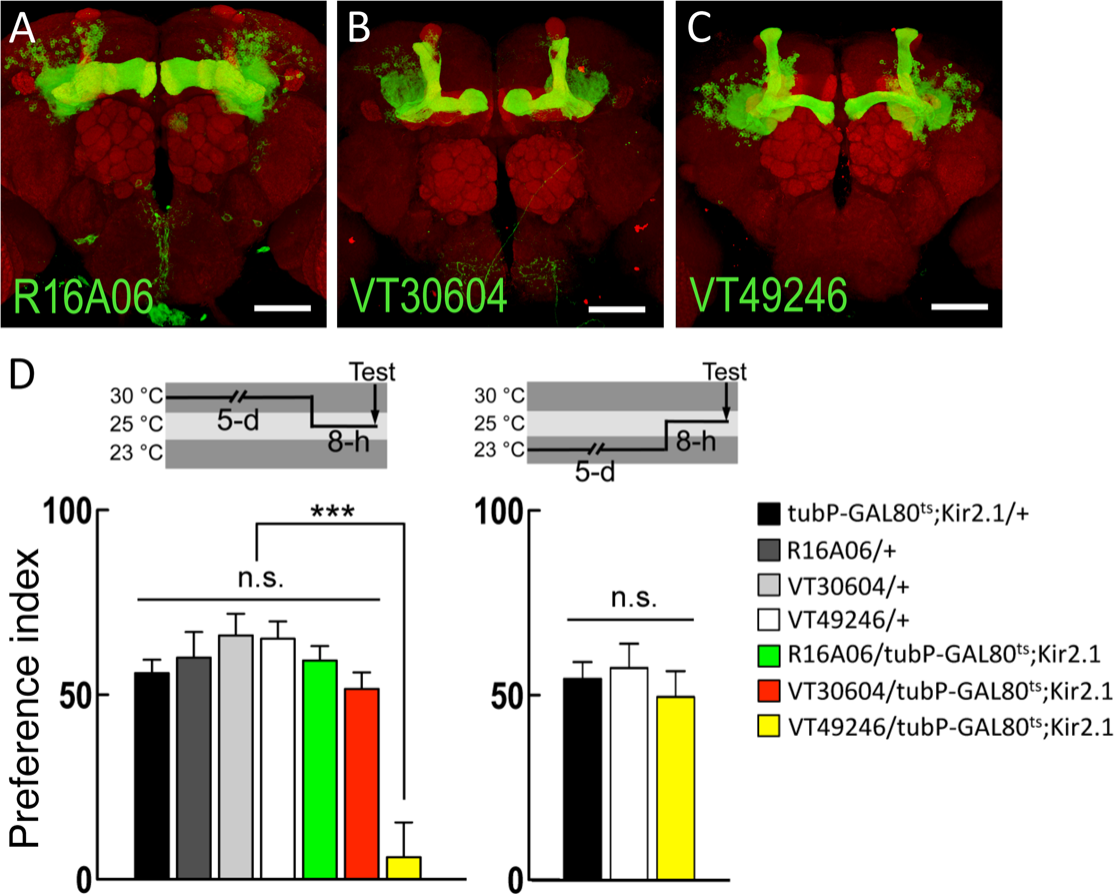
Neural activity in mushroom body αβ neurons is required for normal egg-laying decisions. (A) Preferential expression of *R16A06-GAL4* in mushroom body γ neurons (green). The brain was immunostained with DLG antibody (red). The scale bar represents 50 μm. Genotype was as follow: *UAS-mCD8*::*GFP/+; R16A06-GAL4/UAS-mCD8*::*GFP*. (B) Preferential expression of *VT30604-GAL4* in mushroom body α´β´ neurons (green). The brain was immunostained with DLG antibody (red). The scale bar represents 50 μm. Genotype was as follow: *UAS-mCD8*::*GFP/+; VT30604-GAL4/UAS-mCD8*::*GFP*. (C) Preferential expression of *VT49246-GAL4* in mushroom body αβ neurons (green). The brain was immunostained with DLG antibody (red). The scale bar represents 50 μm. Genotype was as follow: *UAS-mCD8*::*GFP/+; VT49246-GAL4/UAS-mCD8*::*GFP*. (D) Effects of acute silencing of neuronal activity in different mushroom body neuron subsets on egg-laying decisions. The temperature shift protocols are shown schematically above each graph. Each value represents mean ± SEM (n = 10–25, ***P < 0.001. n.s., not statistically significant). Genotypes were as follows: (1) *tubP-GAL80* ^*ts*^ */+; +/UAS-Kir2*.*1*, (2) *+/+; R16A06-GAL4/+*, (3) *+/+; VT30604-GAL4/+*, (4) *+/+; VT49246-GAL4/+*, (5) *tubP-GAL80* ^*ts*^ */+;R16A06-GAL4/UAS-Kir2*.*1*, (6) *tubP-GAL80* ^*ts*^ */+;VT30604-GAL4/UAS-Kir2*.*1*, and (7) *tubP-GAL80* ^*ts*^ */+;VT49246-GAL4/UAS-Kir2*.*1*.

The fibers of DPM neurons innervate the mushroom body, and both axons and dendrites are evenly distributed in the lobes and the anterior peduncle of the mushroom body [6,25]. Therefore, we examined the role of the mushroom body neurons in egg-laying preferences of female flies. The *Drosophila* mushroom body consists of 2000 neurons in each hemisphere of the brain, and the neurons in the mushroom body can be classified into the γ, α´β´, and αβ subsets [8,26]. We examined the effects of acute inhibition of activity in different subsets of mushroom body neurons by *tubP-GAL80* ^*ts*^ *; UAS-Kir2*.*1* combined with *R16A06-GAL4* (γ neurons; Fig. 4A) or *VT30604-GAL4* (α´β´ neurons; Fig. 4B) or *VT49246-GAL4* (αβ neurons; Fig. 4C). Surprisingly, we found that only inhibiting the neuronal activity in the αβ neurons disrupted the normal female egg-laying preference (Fig. 4D). These data suggest that the release of the AMN neuropeptide from DPM neurons onto the mushroom body αβ neurons regulates egg-laying preference in female flies.

## Discussion

The egg-laying site selection by female fruit flies provides a suitable system to study the cellular mechanisms of a simple decision-making behavior [1,2]. When given the direct choice between a sucrose-containing medium and a caffeine-containing medium, flies prefer to lay eggs on the latter. This decision-making process during egg-laying site selection is unchanged in aged animals, suggesting that aging does not dramatically alter the neural activity involved in egg-laying decisions (Fig. 1).

*amn* ^*1*^ is the first *amnesiac* mutant isolated from the behavioral screening for olfactory memory mutants by Quinn et al [5]. Here, we identified the crucial role of the *amn* gene on egg-laying decisions in female flies. The egg-laying preference is altered in *amn* ^*1*^, *amn* ^*28A*^, and *amn* ^*C651*^, and *amn* ^*X8*^ mutants compared to wild-type flies, implying that the *amn* gene product is important for normal egg-laying decisions (Fig. 2). Interestingly, we observed that the *amn* ^*X8*^ showed significant difference in egg-laying preference in sucrose/caffeine or sucrose/plain medium compared to the other *amn* mutants (Figs. 2A and 2B). The original *amn* ^*1*^ is an EMS-induced mutant allele in the *amn* gene while *amn* ^*28A*^ and *amn* ^*c651*^ are P-element-induced mutations in the *amn* gene [5,6,24]. The *amn* ^*X8*^ was made by imprecise excision of the P-element from *amn* ^*28A*^, and a significant increase in ethanol-sensitive phenotype was found in *amn* ^*X8*^ compared to *amn* ^*1*^ and *amn* ^*28A*^ [24]. It is noteworthy that *amn* ^*X8*^ contains possibly other GAL4 insertions elsewhere in the genome left after excision of *amn* ^*28A*^ (Josh Dubnau unpublished data), which may cause a significant negative value of egg-laying preference index in sucrose/caffeine medium (Fig. 2A). Genetic expression of the wild-type *amn* transgene in DPM neurons of *amn* ^*1*^ mutant flies restores the deficiency of egg-laying preference, suggesting that the expression of AMN in DPM neurons is sufficient for normal egg-laying decisions (Fig. 3D). The AMN neuropeptide is a homologue of the vertebrate PACAP that mediates several physiological functions through stimulation of cAMP production [22,27], implying that the cAMP-signaling pathway is important for decision-making processes during egg-laying site selection in *Drosophila*.

Both the axons and dendrites of DPM neurons are evenly distributed in different lobes of the mushroom body, suggesting that DPM neurons receive from and transmit to the mushroom body [25,28]. It has been reported that the neurotransmissions from DPM or mushroom body α´β´ neurons are required for olfactory memory consolidation [13,28]. In addition, the projections of DPM neurons to the α´β´ lobes of the mushroom body are sufficient for stabilizing olfactory memory [28]. These data suggest the possible reciprocal feedback circuits between DPM-mushroom body α´β´ neurons for olfactory memory consolidation [13,28,29]. Our data indicate that AMN release from DPM neurons is critical for normal egg-laying decisions. Silencing the activity in mushroom body αβ neurons also affects this behavior, suggesting that the neural circuitry downstream of DPM neurons modulates egg-laying decisions (Figs. 3E and 4D). However, the neural activity in mushroom body α´β´ neurons is not required for normal egg-laying decisions (Fig. 4D), which indicates the involvement of separate subsets of mushroom body neuron during olfactory memory consolidation and egg-laying decisions. In addition to the AMN neuropeptide, it has been shown that DPM neurons also release serotonin (5HT) onto the mushroom body αβ neurons via the action of the 5HT1A receptor [30]. Whether 5HT and the 5HT1A receptor are required for egg-laying decisions is still unknown.

Interestingly, a recent study identified that different subsets of dopaminergic neurons play opposing roles in egg-laying preference on ethanol substrate in a concentration-dependent manner [3]. Neuronal activity in the mushroom body α´β´ neurons and the ellipsoid body R2 neurons is also required for normal egg-laying preference for ethanol in female flies [3]. We speculate that egg-laying decisions on different substrates (i.e. different concentrations of ethanol-containing foods or sucrose/caffeine containing medium) are mediated by independent subsets of mushroom body neurons. Further study is needed to establish the molecular and neural circuits in the mushroom body involved in decision-making processes during egg-laying site selection in *Drosophila*.

## Materials and Methods

### Fly strains

All the fly stocks were raised on standard cornmeal food at 25°C and 70% relative humidity on a 12:12 h light: dark cycle. The “Cantonized” w^1118^ w(CS10) was used as the wild-type control. The *C316-GAL4* (Bloomington stock number: 30830), *amn* ^*1*^ (Bloomington stock number: 5954), *amn* ^*28A*^, *amn* ^*C651*^, *amn* ^*X8*^, and *UAS-amn* ^*+*^ flies have been described previously [6,7,21,24]. *VT64246-GAL4* and *VT30604-GAL4* flies have been described previously [25,30]. *VT6412-GAL4 and VT49246-GAL4* flies were obtained from the Vienna *Drosophila* Resource Center (VDRC), Vienna Tile. The *R16A06-GAL4* (Bloomington stock number: 48709) flies were obtained from Bloomington stock center. The *tubP-GAL80* ^*ts*^ (Bloomington stock number: 7019)*; UAS-Kir2*.*1*, and *UAS-mCD8*::*GFP* (Bloomington stock number: 5137)*; UAS-mCD8*::*GFP* (Bloomington stock number: 5130) flies were gifts from Dr. Ann-Shyn Chiang.

### Whole-mount immunostaining

Fly brain samples were dissected in phosphate-buffered saline (PBS) and fixed in 4% paraformaldehyde for 20 min at room temperature. After fixation, the brain samples were incubated in PBS containing 1% Triton X-100 and 10% normal goat serum (PBS-T) and degassed in a vacuum chamber to expel tracheal air with six cycles (depressurize to 270 mmHg then hold for 10 min). Next, the brain samples were blocked and penetrated in PBS-T at 25°C for 2 h and then incubated in PBS-T containing 1:10 mouse 4F3 anti-discs large (DLG) monoclonal antibody (Developmental Studies Hybridoma Bank, University of Iowa) at 25°C for one day. After washing in PBS-T three times, the samples were incubated in 1:200 biotinylated goat anti-mouse or rabbit IgG (Molecular Probes) at 25°C for one day. Next, brain samples were washed and incubated in 1:500 Alexa Fluor 635 streptavidin (Molecular Probes) at 25°C for one day. After extensive washing, the brain samples were cleared and mounted in *FocusClear* (CelExplorer) for confocal imaging.

### Confocal microscopy

Sample brains were imaged under a Zeiss LSM 700 confocal microscope with a 40X C-Apochromat water-immersion objective lens. To overcome the limited field of view, some samples were imaged twice, one for each hemisphere, with overlaps in between. We then combined the two parallel image stacks into a single dataset with an on-line stitch of ZEN software, using the overlapping region to align the two stacks.

### Behavioral apparatus

First, 2.5 ml of 3% agarose (with 1% v/v of acetic acid and ethanol) was placed into the behavioral chambers (2.4 cm” L X 2.4 cm” W X 1.5 cm” H plastic container). After the 3% hard agarose solidified, 200 μl of sucrose medium (100 mM sucrose in 1% agarose that contains 1% v/v ethanol) and 200 μl of caffeine medium (100 μM of caffeine in 1% agarose that contains 1% v/v ethanol) were added to opposite sides of a chamber. A small gap (0.1 cm) was made to separate two mediums to prevent a diffusion problem between the two mediums. For the sucrose/plain or caffeine/plain assays, 200 μl of sucrose or caffeine medium were added in one sides and 200 μl of plain medium (1% agarose that contains 1% v/v ethanol) were added to opposite sides of the chamber.

### Behavioral assay

We use a protocol modified from that of Yang et al. [1]. Ten virgin females of specific genotypes and 20 male wild-type flies were placed in empty food vials that only contained wet yeast paste for 24 h to allow them to mate. After mating, all the males were removed and three females were transferred into one behavioral chamber for a 2-h egg-laying preference assay. After 2 h, all the females were removed and the number of eggs on each side of the chamber were counted using a light microscope. The calculation of the performance index followed the method described in Yang et al [1]. The preference index is calculated using the following formula: [(number of eggs on caffeine medium – number of eggs on sucrose medium)/ (number of eggs on caffeine medium **+** number of eggs on sucrose medium)]X100. For sucrose/plain chamber assays (Fig. 2B), the preference index is calculated using following formula: [(number of eggs on plain medium – number of eggs on sucrose medium)/(number of eggs on plain medium **+** number of eggs on sucrose medium)]X100. For caffeine/plain chamber assays (Fig. 2C), the preference index is calculated using following formula: [(number of eggs on plain medium – number of eggs on caffeine medium)/(number of eggs on plain medium **+** number of eggs on caffeine medium)]X100.

### Heat shock protocol

For acute *Kir2*.*1* expression with *tubP-GAL80* ^*ts*^, flies were kept at 18°C throughout development. After eclosion, virgin female flies were collected and housed at 23°C or 30°C for 5 days. On the 4^th^ day, 10 virgin females and 20 male wild-type flies were placed in empty food vials that only contained wet yeast paste for 1 day at either 23°C or 30°C to allow them to mate. After mating, the female flies were separated from males, housed at 25°C for an 8-h incubation and then transferred into the behavioral chamber for the 2-h egg-laying preference assay at 25°C.

### Statistics

All the raw data were analyzed parametrically with JMP5.1 statistical software (SAS Institute Inc.). The data were evaluated by one- or two-way ANOVAs, except for the two-group comparisons in Figs. 1B, 2A (+/+ and *amn* ^*X8*^), and 2B (+/+ and *amn* ^*X8*^), for which paired t-tests were used. Subsequent pairwise planned comparisons were adjusted for experiment-wise error (α), keeping the overall α at 0.05. All data are presented as the mean ± SEM.
